# PCIM-DTA: pairwise conditional interaction modeling for drug–target affinity prediction under cold-start scenarios

**DOI:** 10.1093/bioinformatics/btag646

**Published:** 2026-08-31

**Authors:** Ziyou Zhou, Min Chen, Wenjian Zhou, Qiong Xiao

**Affiliations:** School of Computer Science and Engineering, Hunan Institute of Technology, Hengyang, Hunan 421002, China; Hunan Chuanshan Electronic Technology Co., Ltd, Hengyang, Hunan 421002, China; School of Computer Science and Engineering, Hunan Institute of Technology, Hengyang, Hunan 421002, China; School of Computer Science and Engineering, Hunan Institute of Technology, Hengyang, Hunan 421002, China; School of Computer Science and Engineering, Hunan Institute of Technology, Hengyang, Hunan 421002, China

## Abstract

**Motivation:**

Cold-start drug–target affinity prediction remains challenging because static interaction mechanisms cannot adapt to individual drug–target pairs.

**Results:**

We propose PCIM-DTA, which constructs pair-level interaction representations and derives a pair-specific condition vector from global drug and target features. The condition vector modulates attention, pair-token features, distribution-aware recalibration, and regression parameters, while graph message passing captures higher-order dependencies. Experiments on Davis and BindingDB-Kd show that PCIM-DTA achieves competitive or superior performance under Warm, Cold-drug, Cold-target, Cold-both, and Scaffold-drug settings. Ablation studies support the contribution of each component.

**Availability and Implementation:**

The datasets used in this study are publicly available, including the Davis and BindingDB-Kd datasets. The implementation code of PCIM-DTA is publicly available at https://github.com/1322469934/PCIM-DTA.

## 1 Introduction

Drug–target affinity (DTA) prediction is a fundamental task in computer-aided drug design. It estimates the binding strength between small-molecule drugs and protein targets to support drug screening and optimization (He *et al.* 2025, Shah *et al.* 2025, Zhu *et al.* 2025, Lin *et al.* 2026, Pandey *et al.* 2026).

In practice, models must often predict affinities for unseen drugs or targets, creating cold-start scenarios (Jiang *et al.* 2026, Rakib *et al.* 2026, Tian *et al.* 2026). Cold-start DTA prediction methods can be broadly grouped into representation enhancement-based, data and distribution modeling-based, and interaction modeling-based methods. Cold-start DTA prediction commonly involves unseen drug (cold-drug) and unseen target (cold-target) tasks (Lu *et al.* 2025, 2026, Zhao *et al.* 2025).

Representation enhancement-based methods improve unimodal drug or protein representations to reduce distribution shift in cold-start scenarios (Hu *et al.* 2025, Li *et al.* 2025, Quan *et al.* 2025). For example, Ye and Sun (2024) used graph neural network pre-training to learn molecular and protein structural representations, improving model generalization. Liu *et al.* (2026) proposed DTAP, which combines pre-trained representations with multi-scale structural information to improve performance on unseen samples. Recent studies have also combined protein language models with graph representations to improve DTA prediction (Debnath *et al.* 2025). Zhang *et al.* (2026) enhanced feature representation through multimodal fusion. Wu *et al.* (2024) further combined multimodal graph representations with attention mechanisms to model drug and target features. Despite these advances, the prediction process still relies on independently learned representations. It lacks conditional interaction modeling for specific drug–target pairs, limiting its ability to capture complex interactions.

Data- and distribution-modeling methods alleviate data scarcity and distribution shifts through data augmentation, generative modeling, or transfer learning (He *et al.* 2024, Liu *et al.* 2024). Nguyen *et al.* (2022) incorporated external biological relationship knowledge through cross-task transfer learning to address label scarcity in cold-start scenarios. Kim *et al.* (2025) proposed MixingDTA, which generates virtual samples by mixing embeddings to increase data diversity. Kalemati *et al.* (2024) used generative adversarial networks to construct pseudo-samples and expand the training distribution. Qiao *et al.* (2024) enriched data distribution representations through graph generation and multi-source information fusion. Recent studies have also used diffusion models, such as the Co-Diffusion framework, to learn smooth and generalizable latent distributions (Qian *et al.* 2026). Although these methods alleviate distribution shifts from a data perspective, their improvements largely depend on data augmentation. They do not directly model the drug–target interaction process or its conditional dependencies.

Interaction-modeling methods explicitly characterize drug–target interactions through attention mechanisms or multimodal fusion (Kang *et al.* 2025, Wang *et al.* 2025). Recent studies have also improved DTA prediction through multi-scale diffusion, graph optimization, and associative learning. Representative methods include multi-scale diffusion and interactive learning (Zhu *et al.* 2024), TDGraphDTA (Zhu *et al.* 2023), and an associative learning mechanism (Zhu *et al.* 2023). These methods improve drug–target representations or interaction learning. In contrast, PCIM-DTA focuses on pair-specific conditional interaction modulation under cold-start settings. Zhao *et al.* (2023) proposed AttentionDTA, which learns pair-level interaction representations through attention mechanisms. Li *et al*. (2024) proposed TEFDTA, which jointly models molecular and protein features with Transformers to capture long-range dependencies. MDM-DTA and GPCNDTA (Zhang *et al.* 2023, Dai *et al.* 2026) enhance interaction modeling through cross-modal attention and graph structure modeling. Graph Transformer-based methods (Rakib *et al.* 2026) further model interaction information at multiple scales. However, these methods typically use the same parameter-shared interaction function for different drug–target pairs. They therefore lack sample-specific conditional regulation and cannot readily capture changes in pair-level interaction relationships.

Existing methods have addressed cold-start prediction through representation learning, data augmentation, and interaction modeling. However, most still rely on static modeling paradigms and lack conditional dependency modeling for individual drug–target pairs. Their interaction processes cannot be dynamically adjusted to sample characteristics. Existing methods also tend to model interactions at a single level, without coordinated regulation of attention allocation, feature responses, and data distributions. These limitations restrict the modeling of complex interaction patterns and become more pronounced under cold-start settings, where distribution shifts reduce generalization performance.

To address these issues, we propose PCIM-DTA, which uses a pair-specific condition vector to adapt attention, feature responses, and representation distributions for each drug–target pair. Its main contributions are: (i) a pairwise conditional interaction framework for sample-specific modeling; (ii) coordinated modulation through conditional attention bias, feature gating, and distribution-aware recalibration; and (iii) extensive validation under multiple cold-start settings, demonstrating strong generalization.

## 2 Methodology

As shown in Fig. 1, PCIM-DTA consists of four components: representation learning, pairwise interaction construction, interaction modeling, and prediction.

**Figure 1:**
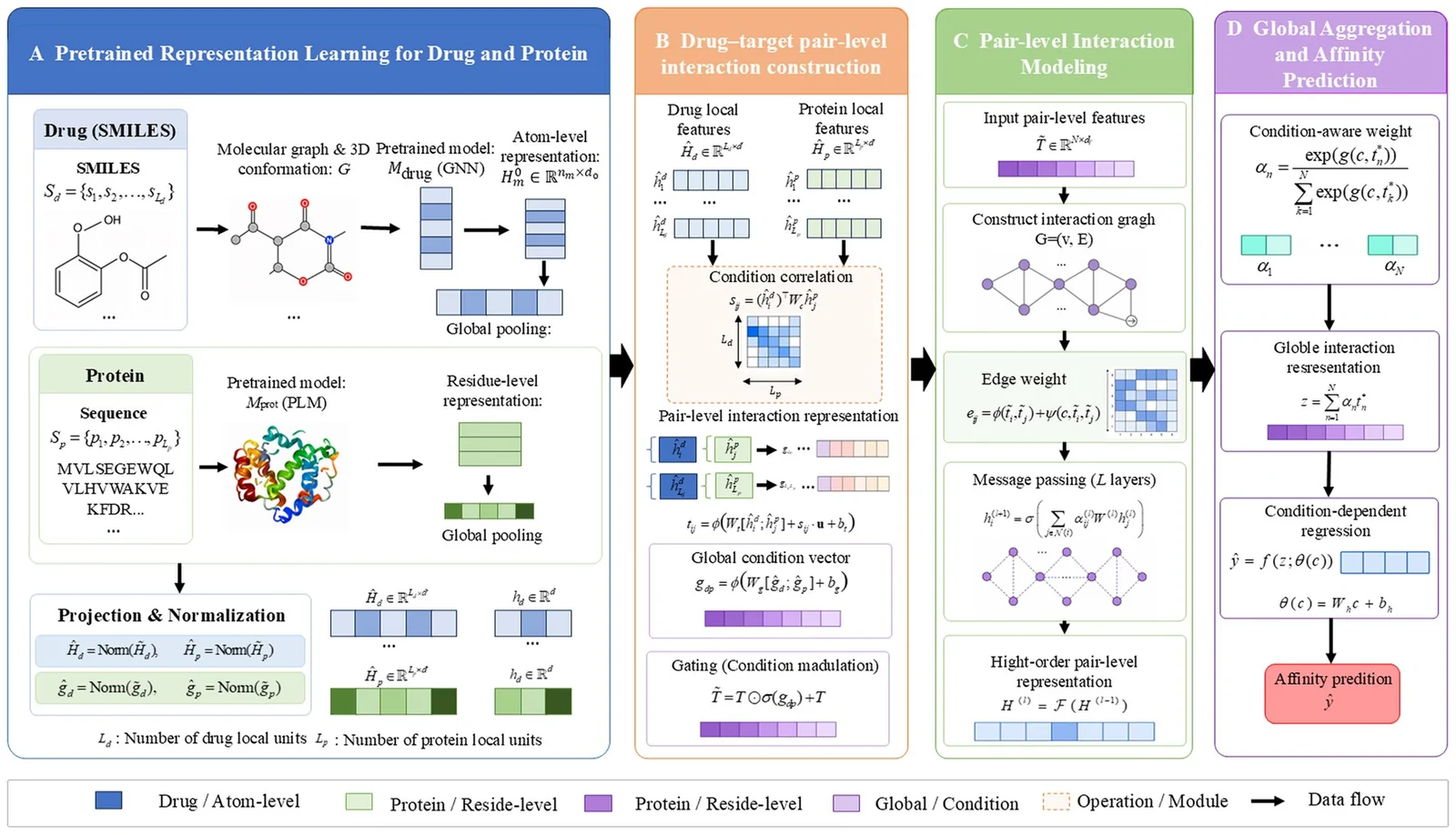
Architecture of the proposed PCIM-DTA framework.

### 2.1 Pre-trained representation learning for drugs and proteins

Pre-trained foundation models are used to obtain drug and protein representations that capture semantic and structural information. The drug SMILES sequence and the protein amino acid sequence are denoted by:


(1) $$S_{d}=\{s_{1},s_{2},\ldots ,s_{L_{d}}\},S_{p}=\{p_{1},p_{2},\ldots ,p_{L_{p}}\}$$

where $L_{d}$ and $L_{p}$ denote the drug and protein sequence lengths, respectively.

For proteins, a pre-trained protein language model encodes the sequence into residue-level contextual representations:


(2) $$H_{p}=E_{p}(S_{p})\in R^{L_{p}\times d_{p}}$$

For drugs, the SMILES sequence is converted into a molecular graph with a three-dimensional conformation, $G_{d}$ ​. A pre-trained molecular model then encodes $G_{d}$ into atom-level representations:


(3) $$H_{d}=E_{d}(G_{d})\in R^{N_{d}\times d_{d}}$$

where $N_{d}$ is the number of atoms. The resulting protein and drug representations capture protein contextual dependencies and molecular topological and geometric information.

Global representations are obtained by pooling the local features:


(4) $$g_{d}=\text{Pool}(H_{d}),\;g_{p}=\text{Pool}(H_{p})$$

where $g_{d},g_{p}\in R^{d}$, and Pool(⋅) denotes the pooling operation. To reduce modality-specific feature discrepancies, the local and global features are projected into a unified feature space:


(5) $${\tilde{H}}_{d}=H_{d}W_{d}+b_{d},\;{\tilde{H}}_{p}=H_{p}W_{p}+b_{p}$$

(6) $${\tilde{g}}_{d}=g_{d}W_{d}+b_{d},\;{\tilde{g}}_{p}=g_{p}W_{p}+b_{p}$$

where $W_{d},W_{p}\in R^{d\times d^{'}}$ are learnable projection matrices, $b_{d}$and $b_{p}$are bias terms, and $d^{'}$is the projected feature dimension.

The projected features are then normalized:


(7) $${\hat{H}}_{d}=\text{Norm}({\tilde{H}}_{d}),\;{\hat{H}}_{p}=\text{Norm}({\tilde{H}}_{p})$$

(8) $${\hat{g}}_{d}=\text{Norm}({\tilde{g}}_{d}),\;{\hat{g}}_{p}=\text{Norm}({\tilde{g}}_{p})$$

where Norm(⋅) denotes the normalization operation.

The normalized local representations ${\hat{H}}_{d}$and ${\hat{H}}_{p}$ are used to construct drug–target pair-level interactions. The global representations $\hat{g}$ and ${\hat{g}}_{p}$ provide semantic information for subsequent interaction modeling.

### 2.2 Pair-level interaction construction

Most existing methods fuse only global drug and protein representations and may overlook fine-grained relationships between local units. We construct pair-level interaction representations by explicitly modeling dependencies between local drug and protein units.

The normalized local representations from Section 2.1 are:


(9) $${\hat{H}}_{d}={\left[{\hat{h}}_{1}^{d},{\hat{h}}_{2}^{d},\ldots ,{\hat{h}}_{L_{d}}^{d}\right]}^{⊤}\in R^{L_{d}\times d^{\prime }}$$

(10) $${\hat{H}}_{p}={\left[{\hat{h}}_{1}^{p},{\hat{h}}_{2}^{p},\ldots ,{\hat{h}}_{L_{p}}^{p}\right]}^{⊤}\in R^{L_{p}\times d^{\prime }}$$

where $L_{d}$ and $L_{p}$ denote the numbers of local drug and protein units, respectively, and $d^{'}$ is the unified feature dimension.

For each pair of local drug and protein units, a condition-aware relevance score is computed as:


(11) $$s_{ij}={\left({\hat{h}}_{i}^{d}\right)}^{⊤}W_{c}{\hat{h}}_{j}^{p}$$

where $W_{c}\in R^{d^{'}\times d^{'}}$ is a learnable matrix, and $s_{ij}$ measures the relevance between the *i*-th drug unit and the *j*-th protein unit.

The corresponding pair-level interaction feature is generated by:


(12) $$t_{ij}=\phi (W_{t}[{\hat{h}}_{i}^{d};{\hat{h}}_{j}^{p}]+s_{ij}\cdot u+b_{t})$$

where $W_{t}\in R^{2d^{'}\times d_{t}}$, $u\in R^{d_{t}}$, and $b_{t}\in R^{d_{t}}$ are learnable parameters. Here, ϕ(⋅) is a nonlinear activation function, and $d_{t}$ is the pair-level feature dimension.

The pair-level interaction feature set is obtained by enumerating all local combinations:


(13) $$T=\text{Stack}(\{t_{ij}\})\in R^{(L_{d}L_{p})\times d_{t}}$$

where each row of *T* represents one drug–protein local interaction.

A global condition vector is constructed to incorporate global matching semantics:


(14) $$g_{dp}=\phi (W_{g}[{\hat{g}}_{d};{\hat{g}}_{p}]+b_{g})$$

where $W_{g}\in R^{2d^{'}\times d_{t}}$, $b_{g}\in R^{d_{t}}$, and $g_{dp}\in R^{d_{t}}$.

The global condition vector is defined as ​*c*=*g_dp_*​. Derived from the global drug and target representations, *c* provides shared conditional information for interaction modeling, conditional gating, distribution-aware recalibration, and the condition-dependent regression head.

The pair-level features are then modulated by the global condition vector:


(15) $$\tilde{T}=T⊙\sigma (g_{dp})+T$$

where σ(⋅) is the sigmoid function and ⊙ denotes element-wise multiplication.

The resulting features encode condition-aware local interactions while retaining global semantic guidance for subsequent interaction modeling.

Let *N_d_* and *L_p_* denote the numbers of drug atoms and protein residues, respectively. The number of pair tokens is *N*=*N_d_ L_p_*, and pair-token construction has a complexity of *O*(*N_d_L_p_d_t_*). To limit computation for long proteins, each protein sequence is truncated to 1022 residues. Before graph interaction, the top-*k* pair tokens are retained based on their pair relevance scores. In our experiments, *k* was selected from {32,64,128} based on validation-set performance. We used *k *= 64 for all reported results. The test set was not used to select *k*.

### 2.3 Pair-level interaction modeling

After obtaining the pair-level interaction features, we model higher-order dependencies among interaction units to capture complex drug–target binding patterns. Let the pair-token matrix be:


(16) $$T^{*}={\left[t_{1}^{*},t_{2}^{*},\ldots ,t_{N}^{*}\right]}^{⊤}\in R^{N\times d_{t}},N=L_{d}L_{p}$$

(17) $$V={\left\{v_{n}\right\}}_{n=1}^{N},v_{n}=t_{n}^{*}$$

where $T^{*}$ denotes the pair-token matrix, and $t_{n}^{*}$ is the *n*-th pair token. Each pair token is represented as a graph node, with $v_{n}=t_{n}^{*}$. These nodes form an interaction graph *G*= (*V*, *E*).

Graph interaction is performed only on the selected top-k pair tokens. This reduces the graph module complexity from O(N2dt) for the full dense graph to O(k2dt), where k≪N. Edge weights combine feature similarity and conditional semantics:


(18) $$e_{ij}=\phi ({\tilde{t}}_{i},{\tilde{t}}_{j})+\psi (c,{\tilde{t}}_{i},{\tilde{t}}_{j})$$

where ψ(⋅) is a global semantic relation function modulated by the condition vector c. Thus, *e_ij_* depends jointly on the similarity between the two pair tokens and *c*. The attention coefficient *α_ij_* is obtained by normalizing *e_ij_* and determines each neighboring node’s contribution during message passing. Message passing is performed as:


(19) $$h_{i}^{\left(l+1\right)}=\sigma \left(\sum_{j\in N(i)}\alpha_{ij}^{\left(l\right)}W^{\left(l\right)}h_{j}^{\left(l\right)}\right)$$

where $h_{i}^{\left(0\right)}={\tilde{t}}_{i}$. The attention coefficient is computed by:


(20) $$\alpha_{ij}^{\left(l\right)}=\frac{\;\text{exp}\;\left(g(c,h_{i}^{\left(l\right)},h_{j}^{\left(l\right)})\right)}{\sum_{k\in N(i)}\;\text{exp}\;\left(g(c,h_{i}^{\left(l\right)},h_{k}^{\left(l\right)})\right)}$$

with


(21) $$g(c,h_{i},h_{j})=w^{⊤}\sigma (W_{h}h_{i}+W_{h}h_{j}+W_{c}c+b)$$

Stacking multiple graph interaction layers progressively captures higher-order dependencies:


(22) $$H^{\left(l\right)}=F(H^{\left(l-1\right)}),\;l=1,2,\ldots ,L$$

where $H^{\left(0\right)}=\tilde{T}$. The final pair-level interaction representation is:


(23) $$T^{*}=H^{\left(L\right)}\in R^{N\times d_{t}}$$

This representation combines local interaction structures with global semantic constraints and is used for subsequent affinity prediction.

### 2.4 Global aggregation and affinity prediction

To predict drug–target affinity, the high-level pair-level representations are aggregated into a compact global interaction representation. Let:


(24) $$T^{*}={\left[t_{1}^{*},t_{2}^{*},\ldots ,t_{N}^{*}\right]}^{T}\in R^{N\times d_{t}}$$

A condition-aware weighting function is defined as:


(25) $$g(c,t_{n}^{*})=w^{⊤}\sigma (W_{t}t_{n}^{*}+W_{c}c+b)$$

The normalized weight of each pair token is:


(26) $$\alpha_{n}=\frac{\;\text{exp}(g(c,t_{n}^{*}))}{\sum_{k=1}^{N}\;\text{exp}\;(g(c,t_{k}^{*}))}$$

The global interaction representation is obtained by:


(27) $$z=\sum_{n=1}^{N}\alpha_{n}t_{n}^{*}$$

where $z\in R^{d_{t}}$, and $\alpha_{n}$denotes the contribution of the n-th pair token.

Attention weighting selects relevant pair tokens, whereas conditional gating modulates token-level features. Distribution-aware recalibration operates on the aggregated representation *z* to account for distribution shifts under cold-start settings.

After obtaining *z*, the distribution-aware recalibration module estimates its mean and variance within each mini-batch. The condition vector *c* generates sample-specific scaling and shifting parameters:


(28) $${\tilde{z}}_{i}=\gamma (c_{i})⊙\frac{z_{i}-\mu_{B}}{\sqrt{\sigma_{B}^{2}+\epsilon }}+\beta (c_{i})$$

where $\mu_{B}$ and $\sigma_{B}^{2}$​ denote the mean and variance of the global interaction representations in the current mini-batch, respectively. Mini-batch statistics are used during training, whereas running statistics accumulated during training are used for testing.

Finally, the recalibrated global representation${\tilde{z}}_{i}$ is passed to the conditional regression head to predict the affinity value.


(29) $${\hat{y}}_{i}=f_{\theta (c_{i})}({\tilde{z}}_{i})$$

(30) $$\theta (c)=W_{h}c+b_{h}$$

where *θ*(*c*) denotes regression parameters generated from the condition representation.

Given *M* training samples, the model is optimized by:


(31) $$L=\frac{1}{M}\sum_{m=1}^{M}{\left({\hat{y}}_{m}-y_{m}\right)}^{2}+\lambda ||\Theta {||}_{2}^{2}$$

where *Θ* denotes all learnable parameters, and *λ* is the regularization coefficient.

Conditional gating uses c to modulate pair-token features at the sample level. Distribution-aware recalibration scales and shifts the global interaction representation according to *c*, reducing the distribution gap between the training and cold-start test sets. The condition-dependent regression head uses parameters *θ*(*c*) generated from the condition vector, allowing the prediction function to adapt to each drug–target pair. The definitions and tensor shapes of the main variables are provided in Table S1.

## 3 Experimental setup

### 3.1 Data splits and experimental design

Two benchmark datasets were used: Davis is kinase-focused and contains 68 drugs, 442 targets, and 30 056 drug–target interactions. BindingDB-Kd is more diverse and contains 1630 drugs, 1021 targets, and 49 830 interactions. Drug molecules were represented as molecular graphs with RDKit-generated 3D conformations, whereas proteins were represented by amino acid sequences. Table 1 summarizes the dataset statistics.

**Table 1:** Dataset statistics.

Dataset	Drugs	Targets	Interactions
Davis	68	442	30 056
BindingDB-Kd	1630	1021	49 830

Drug–target affinity prediction is formulated as a supervised regression task. The *K_d_* values in the Davis and BindingDB-*K_d_*​ datasets are converted to *pK_d_* before training and evaluation. When *K_d_* is measured in nM, the conversion is defined as:


(32) $$pK_{d}=9-{\text{log}}_{10}(K_{d}^{\text{nM}})$$

The Warm setting uses an interaction-level random split. Drugs and targets may appear in multiple subsets, but each drug–target interaction appears in only one subset. Davis and BindingDB-Kd use fixed split ratios of approximately 70%/10%/20% and 80.0%/11.4%/8.6%, respectively. All methods use the same split files.

Two strict cold-start settings, Cold-both and Scaffold-drug, are also evaluated. In the Cold-both setting, neither the drugs nor the targets in the test set appear in the training set. In the Scaffold-drug setting, drugs are grouped by their Bemis–Murcko scaffolds, with no scaffold overlap between subsets. Temporal splitting is not used because the datasets lack reliable timestamps.

Table 2 summarizes the interaction counts for each split. Table S2 reports the numbers of unique drugs, unique targets, and interactions in each subset under both standard and strict cold-start settings.

**Table 2:** Statistics of dataset splits under different evaluation settings.

Dataset	Setting	Train	Valid	Test
Davis	Warm	21 038	3006	6012
	Cold-drug	20 774	3094	6188
	Cold-target	20 944	3060	6052
BindingDB-Kd	Warm	39 870	5696	4264
	Cold-drug	36 000	5500	8330
	Cold-target	40 730	4836	4264

Entity leakage was checked after data splitting. The Cold-drug setting satisfies:


(33) $$D_{\text{train}}\cap D_{\text{valid}}=Ø,\;D_{\text{train}}\cap D_{\text{test}}=Ø,\;D_{\text{valid}}\cap D_{\text{test}}=Ø$$

The Cold-target setting satisfies:


(34) $$T_{\text{train}}\cap T_{\text{valid}}=Ø,\;T_{\text{train}}\cap T_{\text{test}}=Ø,\;T_{\text{valid}}\cap T_{\text{test}}=Ø$$

The Warm setting only ensures that no interaction is repeated across subsets. In the Cold-both setting, the drug sets of the training, validation, and test subsets are pairwise disjoint. The corresponding target sets are also pairwise disjoint. In the Scaffold-drug setting, the scaffold groups of the three subsets are pairwise disjoint. All strict cold-start splits are stored as fixed split files and used by all methods.

### 3.2 Baselines and ablation design

The baselines cover sequence-, graph-, and diffusion-based paradigms: DeepDTA (CNN-based sequence modeling) (Öztürk *et al.* 2018), GraphDTA (graph neural network) (Nguyen *et al.* 2021), PMHGT-DTA (multimodal graph Transformer) (Zhang *et al.* 2026), CASTER-DTA (equivariant GNN) (Kumar *et al.* 2025), DTAP (unified graph Transformer) (Liu *et al.* 2026), and Co-Diffusion (latent diffusion) (Qian *et al.* 2026). Official implementations are used when available. Otherwise, the methods are reproduced using the settings reported in their original papers. Each baseline retains its original encoder configuration rather than adopting the encoders used in this work. All methods use the same training, validation, and test splits. The main hyperparameters are selected on the validation set.

The progressive ablation variants include Base1 (independent representations only), Base2 (+pair-level interaction construction), Base3 (+conditional attention bias), Base4 (+conditional feature gating), and the full PCIM-DTA (+distribution-aware recalibration). Four controlled variants are also evaluated: cross-attention without conditional modulation, FiLM-style conditioning, dynamic regression head only, and pairwise interaction without graph message passing. These variants examine the individual effects of conditional modulation, graph message passing, and the dynamic regression head.

Component-wise ablations are provided in the Supplementary Material. They examine the contributions of the condition source, attention bias, feature gating, distribution-aware recalibration, interaction graph, pre-trained drug encoder, pre-trained protein encoder, and condition-dependent regression head.

### 3.3 Implementation details

All experiments were conducted on an NVIDIA RTX 3080 Ti GPU. Table 3 summarizes the main hyperparameters. The model was trained using AdamW (Hospodarskyy *et al.* 2024) with a learning rate of 1 × 10^−4^, weight decay of 1 × 10^−5^, hidden dimension of 256, batch size of 128, dropout rate of 0.1, and a maximum of 100 epochs. Early stopping was applied with a patience of 10 epochs. All baselines used the same training settings and data split files for fair comparison. The learning rate, batch size, dropout rate, and weight decay were selected using only the validation set. The test set was not used for model selection or hyperparameter tuning. Inference runtime and peak GPU memory usage are reported in Table S3. Results are reported as mean ± std over five independent runs.

**Table 3:** Hyper-parameter settings of PCIM-DTA.

Parameter	Value
Drug Encoder	GIN with 3D geometric features
Protein Encoder	ESM-2
Drug Input	Molecular graph + RDKit-generated 3D conformation
Conformer Generation	ETKDG + MMFF94 optimization
Max Protein Length	1022
Hidden Dimension	256
Conditional Network	2-layer FFN (512 units)
Interaction Module	Cross-attention + gating
Batch Size	128
Optimizer	AdamW
Learning Rate	1 × 10^–4^
Weight Decay	1 × 10^–5^
Epochs	100
Early Stopping	Patience = 10
Dropout	0.1
Loss Function	MSE

## 4 Results and analysis

### 4.1 Overall performance

Tables 4 and 5 report the results on Davis and BindingDB-Kd. Figure 2 shows the predicted and true affinity values. Under the Warm setting on Davis, PCIM-DTA achieves the best results across all metrics, with an MSE of 0.188, a CI of 0.921, and an R2 of 0.733. Its MSE is 3.6% lower than that of Co-Diffusion (0.195).

**Figure 2:**
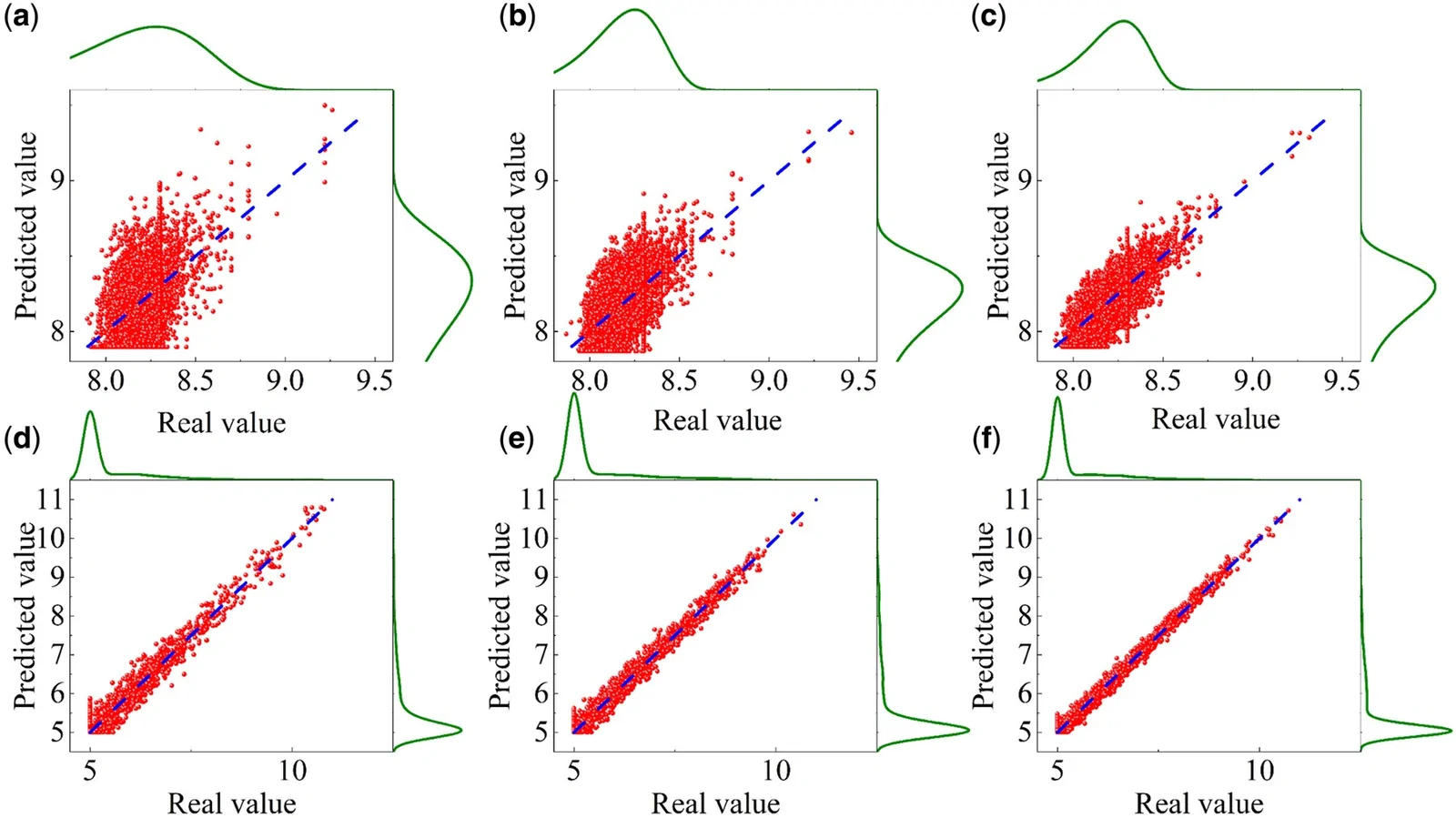
Scatter plots of predicted versus true affinity values for PCIM-DTA under different evaluation settings. (a–c) Davis: Cold-drug, Cold-target, and Warm. (d–f) BindingDB-Kd: Cold-drug, Cold-target, and Warm. The blue dashed line represents the ideal prediction line.

**Table 4:** Performance comparison on the Davis dataset (mean ± standard deviation over 5 runs).

Method	Settings	MSE ↓	CI ↑	R² ↑	Pearson ↑	Spearman ↑
DeepDTA	Warm	0.261 ± 0.0268	0.878 ± 0.0345	0.630 ± 0.0397	0.867 ± 0.0248	0.862 ± 0.0056
GraphDTA		0.239 ± 0.0561	0.883 ± 0.0246	0.661 ± 0.0486	0.875 ± 0.0231	0.866 ± 0.0059
PMHGT-DTA		0.236 ± 0.0334	0.901 ± 0.0242	0.665 ± 0.0248	0.868 ± 0.0145	0.882 ± 0.0054
CASTER-DTA		0.208 ± 0.0249	0.897 ± 0.0337	0.705 ± 0.0269	0.893 ± 0.0252	0.899 ± 0.0037
DTAP		0.200 ± 0.0447	0.901 ± 0.0129	0.716 ± 0.0267	0.904 ± 0.0238	0.901 ± 0.0036
Co-Diffusion		0.195 ± 0.0222	0.917 ± 0.0219	0.724 ± 0.0231	0.910 ± 0.0227	0.903 ± 0.0031
PCIM-DTA		0.188 ± 0.0239	0.921 ± 0.0318	0.733 ± 0.0055	0.918 ± 0.0225	0.914 ± 0.0030
DeepDTA	Cold-drug	0.421 ± 0.0176	0.812 ± 0.0287	0.488 ± 0.0214	0.785 ± 0.0109	0.771 ± 0.0121
GraphDTA		0.367 ± 0.0131	0.847 ± 0.0188	0.554 ± 0.0159	0.812 ± 0.0294	0.801 ± 0.0102
PMHGT-DTA		0.338 ± 0.0116	0.842 ± 0.0168	0.589 ± 0.0141	0.826 ± 0.0186	0.815 ± 0.0097
CASTER-DTA		0.328 ± 0.0112	0.859 ± 0.0172	0.601 ± 0.0136	0.842 ± 0.0284	0.831 ± 0.0089
DTAP		0.301 ± 0.0132	0.868 ± 0.0262	0.634 ± 0.0161	0.851 ± 0.0171	0.841 ± 0.0080
Co-Diffusion		0.291 ± 0.0123	0.880 ± 0.0163	0.646 ± 0.0150	0.862 ± 0.0264	0.853 ± 0.0073
PCIM-DTA		0.253 ± 0.0194	0.896 ± 0.0154	0.692 ± 0.0114	0.881 ± 0.0257	0.872 ± 0.0066
DeepDTA	Cold-target	0.465 ± 0.0193	0.792 ± 0.0196	0.434 ± 0.0235	0.761 ± 0.0124	0.748 ± 0.0135
GraphDTA		0.398 ± 0.0147	0.821 ± 0.0182	0.516 ± 0.0179	0.797 ± 0.0114	0.793 ± 0.0114
PMHGT-DTA		0.372 ± 0.0135	0.836 ± 0.0171	0.547 ± 0.0164	0.811 ± 0.0107	0.789 ± 0.0101
CASTER-DTA		0.345 ± 0.0112	0.852 ± 0.0274	0.580 ± 0.0136	0.827 ± 0.0192	0.815 ± 0.0093
DTAP		0.332 ± 0.0128	0.861 ± 0.0162	0.596 ± 0.0156	0.839 ± 0.0288	0.820 ± 0.0092
Co-Diffusion		0.318 ± 0.0101	0.872 ± 0.0157	0.613 ± 0.0123	0.847 ± 0.0176	0.835 ± 0.0112
PCIM-DTA		0.286 ± 0.0186	0.889 ± 0.0149	0.652 ± 0.0105	0.873 ± 0.0173	0.861 ± 0.0085

**Table 5:** Reports the comparison with representative baseline methods on BindingDB-Kd.

Method	Settings	MSE ↓	CI ↑	R² ↑	Pearson ↑	Spearman ↑
DeepDTA	Warm	0.566 ± 0.0337	0.854 ± 0.0340	0.695 ± 0.0182	0.875 ± 0.0260	0.882 ± 0.0047
GraphDTA		0.605 ± 0.0719	0.849 ± 0.0380	0.674 ± 0.0387	0.869 ± 0.0131	0.872 ± 0.0119
PMHGT-DTA		0.518 ± 0.0599	0.859 ± 0.0472	0.721 ± 0.0323	0.884 ± 0.0104	0.887 ± 0.0094
CASTER-DTA		0.498 ± 0.0298	0.861 ± 0.0443	0.732 ± 0.0161	0.887 ± 0.0265	0.889 ± 0.0066
DTAP		0.443 ± 0.0500	0.870 ± 0.0348	0.761 ± 0.0270	0.900 ± 0.0288	0.902 ± 0.0067
Co-Diffusion		0.453 ± 0.0671	0.867 ± 0.0388	0.756 ± 0.0361	0.896 ± 0.0132	0.897 ± 0.0130
PCIM-DTA		0.419 ± 0.0582	0.869 ± 0.0479	0.774 ± 0.0313	0.904 ± 0.0114	0.904 ± 0.0109
DeepDTA	Cold-drug	0.618 ± 0.0220	0.821 ± 0.0430	0.509 ± 0.0175	0.822 ± 0.0263	0.828 ± 0.0066
GraphDTA		0.555 ± 0.0283	0.827 ± 0.0443	0.559 ± 0.0225	0.832 ± 0.0198	0.835 ± 0.0072
PMHGT-DTA		0.534 ± 0.0932	0.829 ± 0.0127	0.576 ± 0.0740	0.838 ± 0.0236	0.839 ± 0.0216
CASTER-DTA		0.475 ± 0.0487	0.840 ± 0.0466	0.622 ± 0.0387	0.852 ± 0.0138	0.857 ± 0.0118
DTAP		0.457 ± 0.0348	0.843 ± 0.0550	0.637 ± 0.0277	0.857 ± 0.0188	0.861 ± 0.0090
Co-Diffusion		0.463 ± 0.0323	0.836 ± 0.0453	0.632 ± 0.0257	0.854 ± 0.0179	0.854 ± 0.0082
PCIM-DTA		0.438 ± 0.0553	0.845 ± 0.0479	0.652 ± 0.0440	0.863 ± 0.0130	0.865 ± 0.0130
DeepDTA	Cold-target	0.632 ± 0.0407	0.814 ± 0.0443	0.463 ± 0.0346	0.806 ± 0.0167	0.814 ± 0.0070
GraphDTA		0.588 ± 0.0586	0.821 ± 0.0471	0.501 ± 0.0498	0.818 ± 0.0132	0.824 ± 0.0130
PMHGT-DTA		0.539 ± 0.0669	0.824 ± 0.0303	0.542 ± 0.0569	0.826 ± 0.0171	0.830 ± 0.0177
CASTER-DTA		0.580 ± 0.0809	0.821 ± 0.0484	0.507 ± 0.0688	0.819 ± 0.0169	0.823 ± 0.0156
DTAP		0.509 ± 0.0593	0.831 ± 0.0564	0.567 ± 0.0504	0.836 ± 0.0137	0.841 ± 0.0107
Co-Diffusion		0.482 ± 0.0452	0.830 ± 0.0559	0.590 ± 0.0384	0.841 ± 0.0118	0.842 ± 0.0098
PCIM-DTA		0.426 ± 0.0540	0.841 ± 0.0491	0.638 ± 0.0459	0.858 ± 0.0150	0.858 ± 0.0154

The improvement is larger under the cold-start settings. Under Cold-drug, PCIM-DTA achieves an MSE of 0.253, which is 13.1% lower than that of Co-Diffusion (0.291). Under Cold-target, its MSE is 0.286, compared with 0.318 for Co-Diffusion. On BindingDB-Kd, PCIM-DTA also achieves the best results under all three settings, with MSE values of 0.419, 0.438, and 0.426 under Warm, Cold-drug, and Cold-target, respectively. The scatter plots show that the predictions generally cluster around the ideal prediction line across the evaluated settings.

Table 6 compares PCIM-DTA with the controlled variants. PCIM-DTA achieves the lowest MSE in five of the six evaluated settings. Under the BindingDB-Kd Cold-drug setting, FiLM-style conditioning obtains a slightly lower MSE than PCIM-DTA (0.429 vs. 0.438). FiLM-style conditioning performs closest to the full model, whereas the other variants produce higher errors, particularly under cold-start settings. These results indicate that the performance gains are associated with the combined use of pair-level interaction, conditional modulation, graph message passing, and the condition-dependent regression head. Paired t-test results for PCIM-DTA and Co-Diffusion are reported in Table S4.

**Table 6:** Compares PCIM-DTA with four strong controlled variants.

Dataset	Setting	Dynamic head only	Pairwise w/o graph	Cross-attention w/o cond	FiLM-style	PCIM-DTA
Davis	Warm	0.209 ± 0.0519	0.204 ± 0.0445	0.201 ± 0.0515	0.195 ± 0.0441	0.188 ± 0.0239
Davis	Cold-drug	0.324 ± 0.0531	0.312 ± 0.0568	0.302 ± 0.0527	0.276 ± 0.0436	0.253 ± 0.0194
Davis	Cold-target	0.360 ± 0.0609	0.344 ± 0.0534	0.334 ± 0.0529	0.306 ± 0.0514	0.286 ± 0.0186
BindingDB-Kd	Warm	0.468 ± 0.0576	0.456 ± 0.0509	0.449 ± 0.0556	0.438 ± 0.0450	0.419 ± 0.0582
BindingDB-Kd	Cold-drug	0.486 ± 0.0554	0.468 ± 0.0437	0.459 ± 0.0545	0.429 ± 0.0541	0.438 ± 0.0553
BindingDB-Kd	Cold-target	0.522 ± 0.0534	0.502 ± 0.0501	0.491 ± 0.0538	0.458 ± 0.0442	0.426 ± 0.0540

Table 7 reports the results under the Cold-both and Scaffold-drug settings. PCIM-DTA achieves the lowest MSE in both settings on both datasets. The errors are higher than those under the standard Cold-drug and Cold-target settings, indicating that Cold-both and Scaffold-drug impose stricter extrapolation conditions.

**Table 7:** Results under stricter cold-start settings based on MSE.

Dataset	Setting	DeepDTA	GraphDTA	Co-Diffusion	FiLM-style	PCIM-DTA
Davis	Cold-both	0.512 ± 0.0535	0.446 ± 0.0687	0.371 ± 0.0599	0.352 ± 0.0568	0.334 ± 0.0449
Davis	Scaffold-drug	0.468 ± 0.0667	0.405 ± 0.0579	0.342 ± 0.0578	0.325 ± 0.0572	0.309 ± 0.0548
BindingDB-Kd	Cold-both	0.702 ± 0.0608	0.641 ± 0.0583	0.522 ± 0.0563	0.501 ± 0.0559	0.481 ± 0.0574
BindingDB-Kd	Scaffold-drug	0.664 ± 0.0587	0.604 ± 0.0575	0.496 ± 0.0480	0.474 ± 0.0575	0.456 ± 0.0593

### 4.2 Ablation study

Figure 3 shows the ablation results under the Cold-target setting. On Davis, the MSE decreases from 0.356 for Base1 to 0.328 for Base2, 0.305 for Base3, 0.292 for Base4, and 0.286 for the full model. On BindingDB-Kd, it decreases from 0.655 to 0.426. These results indicate complementary contributions from the added modules, with a larger overall improvement on BindingDB-Kd.

**Figure 3:**
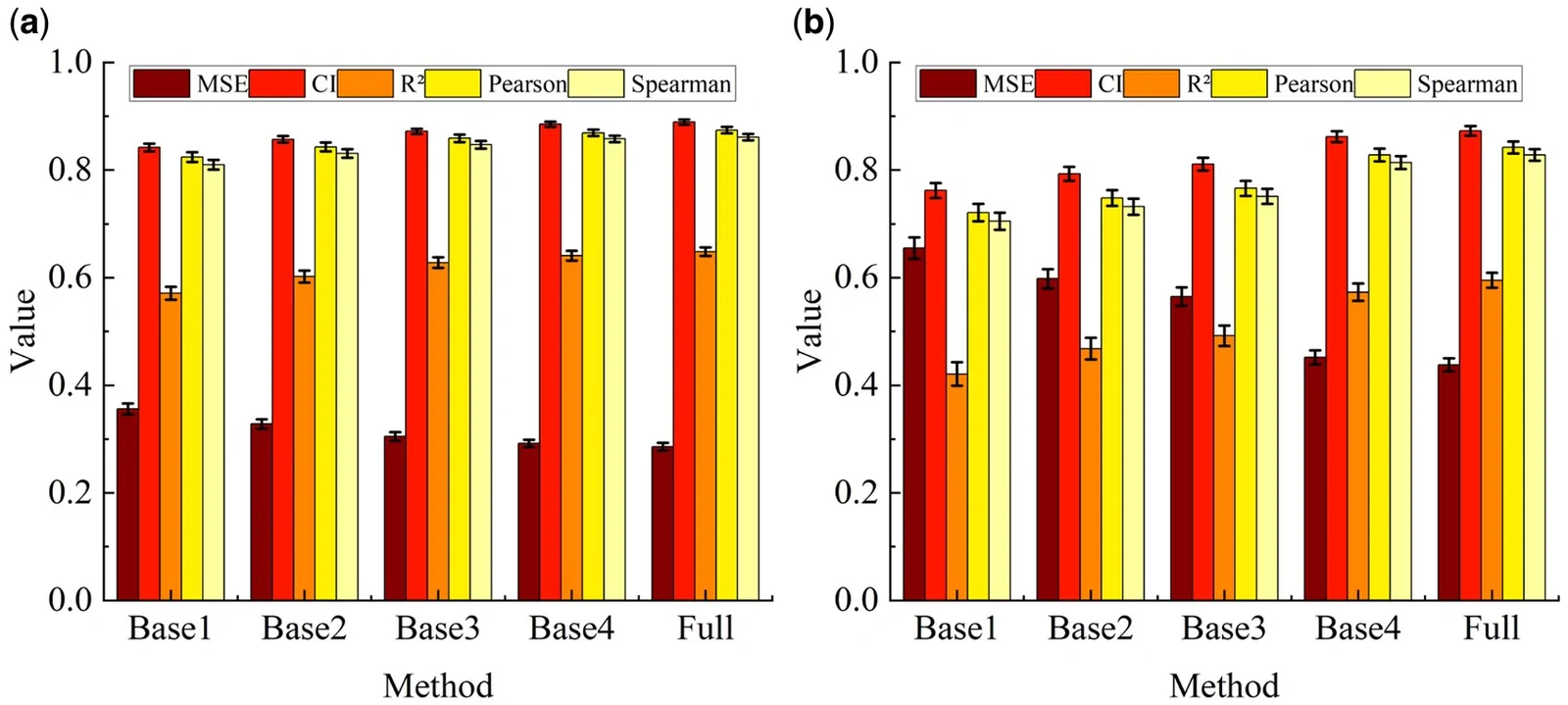
Ablation study results of PCIM-DTA under the cold-target setting. (a) Results on the Davis dataset. (b) Results on the BindingDB-Kd dataset.

Across most settings, the full PCIM-DTA achieves lower MSE than the controlled variants. An exception is the BindingDB-Kd Cold-drug setting, where FiLM-style conditioning achieves a slightly lower MSE. Considering the overlapping standard deviations, the two variants show comparable performance in this setting. This supports the complementary roles of conditional modulation, graph message passing, and the condition-dependent regression head.

Table S5 shows that removing any key component increases the MSE. The largest increases follow the removal of the pair-specific condition, interaction graph, or pre-trained protein encoder.

Table S6 shows that distribution-aware recalibration reduces the representation gap between the training and cold-start test sets. The recalibrated representations have lower MMD, mean gap, and covariance gap than the original representations *z*.

### 4.3 Case study

Table 8 reports predictions for representative drug–target pairs. PCIM-DTA achieves the smallest absolute error in each case: 0.03 for Imatinib–ABL1, 0.04 for Erlotinib–EGFR, and 0.07 for both BindingDB-Kd samples. These errors are lower than those of GraphDTA and Co-Diffusion, indicating more accurate case-level predictions.

**Table 8:** Case-level prediction performance on representative drug–target pairs.

Complex	Dataset	True	GraphDTA	Co-Diffusion	PCIM-DTA	Abs Error (Ours) ↓
Imatinib–ABL1	Davis	7.21	6.82	7.05	7.18	0.03
Erlotinib–EGFR	Davis	7.35	6.94	7.20	7.31	0.04
Sorafenib–RAF1	Davis	7.05	6.61	6.88	7.00	0.05
Dasatinib–SRC	BindingDB	8.12	7.56	7.88	8.05	0.07
Gefitinib–EGFR	BindingDB	7.98	7.42	7.75	7.91	0.07

Table 9 presents the top-10 candidate drugs predicted for EGFR. Most of the highly ranked compounds are approved or clinically investigated EGFR inhibitors, including gefitinib, erlotinib, afatinib, dacomitinib, and osimertinib. This result indicates that PCIM-DTA can assign high affinity scores to compounds with established EGFR-related activity. However, the ranking is used only for retrospective evaluation and biological plausibility analysis, rather than as evidence of prospective drug discovery.

**Table 9:** Top-10 predicted candidate drugs for a target protein (EGFR).

Rank	Drug name	DrugBank ID	Predicted affinity	Evidence
1	Gefitinib	DB00317	8.02	Approved
2	Erlotinib	DB00530	7.95	Approved
3	Afatinib	DB08916	7.89	Approved
4	Lapatinib	DB01259	7.75	Clinical
5	Vandetanib	DB06220	7.68	Literature
6	Dacomitinib	DB11963	7.63	Approved
7	Osimertinib	DB09330	7.58	Approved
8	Neratinib	DB11828	7.52	Clinical
9	Pelitinib	DB12001	7.45	Literature
10	Canertinib	DB05030	7.40	Literature

Note: The top-ranked compounds are known EGFR-related inhibitors. They were retained for retrospective ranking evaluation and biological plausibility analysis, not as independent evidence of prospective discovery.

To further examine the structural plausibility of the predicted interactions, molecular docking was performed using the EGFR–gefitinib co-crystal structure (PDB ID: 4WKQ). As shown in Fig. 4, the docked pose of gefitinib closely overlaps with its co-crystallized conformation in the ATP-binding pocket. Ligands were prepared using RDKit 3D conformer generation and MMFF94 optimization, and docking was conducted with AutoDock Vina 1.2.x. The grid box was centered on the co-crystallized ligand and covered the ATP-binding pocket. Redocking was considered successful when the heavy-atom RMSD was below 2.0 Å. Detailed docking parameters and data-overlap checks are provided in Table S7.

**Figure 4:**
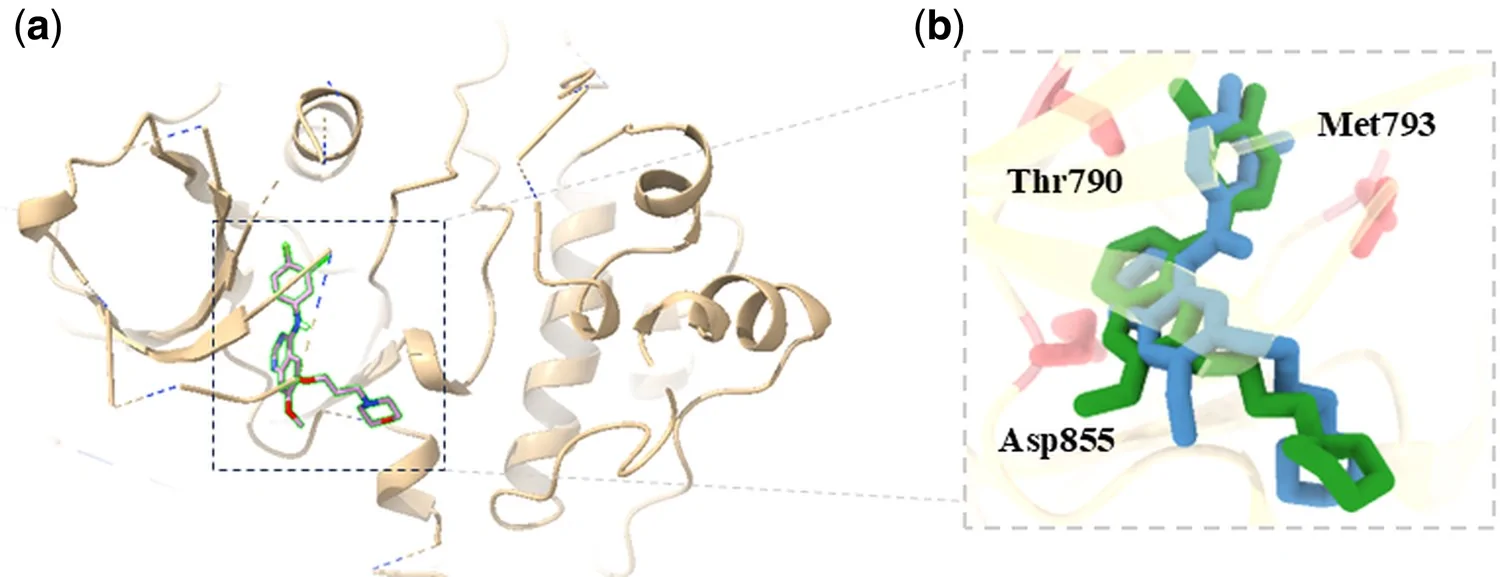
Structural comparison of the docked and co-crystallized ligand conformations in the EGFR binding pocket. (a) Overall protein–ligand complex, with the docked pose (blue) superimposed on the co-crystallized ligand (green). (b) Enlarged view of the binding pocket showing the conformational overlap and the key residues Thr790, Met793, and Asp855.

The learned interaction weights were also mapped to ligand atoms and EGFR residues. As reported in Table S8 and illustrated in Fig. 4, the top-weighted pair tokens are mainly located within or near the ATP-binding pocket, including Met793, Thr790, Leu788, Leu792, and Asp855. This correspondence suggests that the learned pair-level weights are biologically consistent with known EGFR binding regions. Nevertheless, this retrospective case study does not establish causal interpretability or prospective discovery capability.

## 5 Discussion

PCIM-DTA generally performs best under cold-start settings, except for BindingDB-Kd Cold-drug, where FiLM-style achieves a slightly lower MSE. Overall, the results support the effectiveness of explicit conditional interaction modeling under distribution shift. Conditional modulation adjusts features for each drug–target pair rather than relying on fixed mappings. This design is associated with a smaller performance decline from Warm to Cold settings. Case-level analyses also show accurate predictions and biological consistency for representative samples.

The performance gains arise from three complementary design elements. First, pair-level conditional representations address the limitations of static fusion. Second, modulation at the attention, feature, and distribution levels captures interaction patterns at different granularities. Third, the condition-dependent regression head adapts the prediction function to each drug–target pair.

Although PCIM-DTA improves cold-start drug–target affinity prediction, several limitations remain. The model does not explicitly incorporate protein 3D structures or binding pocket annotations during training, which limits mechanistic interpretability. The EGFR case study provides a retrospective biological consistency check. The learned interaction weights serve only as auxiliary evidence and should not be interpreted as causal explanations of binding. Additional external validation on benchmarks such as KIBA is also needed. PCIM-DTA incurs higher computational costs than simpler fusion-based models in large-scale virtual screening. Top-*k* pair-token selection reduces this cost, but further optimization is required. Future work will incorporate protein structural priors, broaden external benchmark validation, and develop more rigorous atom–residue attribution methods.

## 6 Conclusion

We propose PCIM-DTA for drug–target affinity prediction under cold-start scenarios. PCIM-DTA treats drug–target pairs as the basic modeling unit and applies conditional modulation at the attention, feature, and distribution levels. This design adapts interaction representations to each drug–target pair. Experiments on Davis and BindingDB-Kd show consistent improvements over representative baselines across all evaluation settings. Ablation results confirm the contribution of each modulation component. Future work will further integrate structural priors and large-scale pre-trained models to improve interaction modeling.

## Supplementary Material

btag646_Supplementary_Data

## Data Availability

The datasets used in this study are publicly available, including the Davis and BindingDB-Kd datasets. The implementation code is publicly available at https://github.com/1322469934/PCIM-DTA.
