# Supplementary material for: PCIM-DTA: pairwise conditional interaction modeling for drug–target affinity prediction under cold-start scenarios
Source: Bioinformatics. 2026 Aug 31;42(9):btag646. doi: 10.1093/bioinformatics/btag646 (PMC13662694; doi:10.1093/bioinformatics/btag646)
Supplement: Supplementary file 1 [file btag646_supplementary_data.docx]

Supplementary Table S1. Tensor shapes of major variables in PCIM-DTA.

| Symbol | Meaning | Tensor shape |
| --- | --- | --- |
| *H_d_* | drug local representations | *L_d_×d'* |
| *H_p_* | protein local representations | *L_p_×d'* |
| *g_d_* | drug global representation | *d'* |
| *g_p_* | protein global representation | *d'* |
| *c=g_dp_* | pair-specific global condition vector generated from *g_d_* and *g_p_* | *d_t_* |
| *T* | initial pair-token matrix | *N ×d_t_* |
| *T^*^* | modulated pair-token matrix | *N ×d_t_* |
| *v_n_* | graph node / pair token | *d_t_* |
| *e_ij_* | edge weight between nodes | scalar |
| *α_ij_* | attention coefficient | scalar |
| *z* | global interaction representation | *d_t_* |
| *θ_c_* | dynamic regression parameters | task-dependent |
| $\hat{y}$ | predicted affinity | scalar |

Supplementary Table S2. Entity-level statistics of all train/validation/test splits.

| Dataset | Split group | Setting | Subset | Drugs | Targets | Interactions |
| --- | --- | --- | --- | --- | --- | --- |
| Davis | Standard | Warm | Train | 68 | 442 | 21,038 |
|  |  | Warm | Validation | 68 | 440 | 3,006 |
|  |  | Warm | Test | 68 | 442 | 6,012 |
|  |  | Cold-drug | Train | 54 | 442 | 20774 |
|  |  | Cold-drug | Validation | 7 | 438 | 3,094 |
|  |  | Cold-drug | Test | 7 | 441 | 4188 |
|  |  | Cold-target | Train | 68 | 345 | 20944 |
|  |  | Cold-target | Validation | 66 | 42 | 3060 |
|  |  | Cold-target | Test | 67 | 55 | 6052 |
| BindingDB-Kd |  | Warm | Train | 1,628 | 1,021 | 39,870 |
|  |  | Warm | Validation | 1,564 | 1,008 | 5,696 |
|  |  | Warm | Test | 1,512 | 1,002 | 4,264 |
|  |  | Cold-drug | Train | 1,170 | 1,021 | 36,000 |
|  |  | Cold-drug | Validation | 180 | 986 | 5,500 |
|  |  | Cold-drug | Test | 280 | 1,004 | 8,330 |
|  |  | Cold-target | Train | 1,588 | 835 | 40,730 |
|  |  | Cold-target | Validation | 1,420 | 95 | 4,836 |
|  |  | Cold-target | Test | 1,395 | 91 | 4,264 |
| Davis | Strict | Cold-both | Train | 54 | 354 | 19,116 |
|  |  | Cold-both | Validation | 7 | 44 | 308 |
|  |  | Cold-both | Test | 7 | 44 | 308 |
|  |  | Scaffold-drug | Train | 52 | 442 | 22,984 |
|  |  | Scaffold-drug | Validation | 8 | 442 | 3,536 |
|  |  | Scaffold-drug | Test | 8 | 442 | 3,536 |
| BindingDB-Kd |  | Cold-both | Train | 1,170 | 815 | 28,700 |
|  |  | Cold-both | Validation | 180 | 102 | 1,050 |
|  |  | Cold-both | Test | 280 | 104 | 2,350 |
|  |  | Scaffold-drug | Train | 1,130 | 1,021 | 34,500 |
|  |  | Scaffold-drug | Validation | 200 | 990 | 6,400 |
|  |  | Scaffold-drug | Test | 300 | 1,004 | 8,930 |
| Note: Standard includes Warm, Cold-drug, and Cold-target settings; Strict includes Cold-both and Scaffold-drug settings. In Cold-both, mixed-block interactions were not used for training, validation, or testing to avoid double cold-start leakage. | | | | | | |

Supplementary Table S3. Runtime and memory comparison in virtual screening.

| Method | Avg. inference time / 1,000 pairs | Peak GPU memory | Notes |
| --- | --- | --- | --- |
| GraphDTA | 14.8 s | 3.2 GB | graph encoder |
| PMHGT-DTA | 22.5 s | 5.6 GB | Transformer-based |
| Co-Diffusion | 31.7 s | 6.4 GB | diffusion module |
| PCIM-DTA | 26.9 s | 5.9 GB | top-(k) pair tokens |

Supplementary Table S4. Paired t-test between PCIM-DTA and Co-Diffusion based on MSE.

| Dataset | Setting | Co-Diffusion MSE | PCIM-DTA MSE | ΔMSE | 95% CI of ΔMSE | p-value |
| --- | --- | --- | --- | --- | --- | --- |
| Davis | Warm | 0.197 ± 0.0022 | 0.188 ± 0.0239 | -0.009 | [-0.012, -0.006] | 0.003 |
| Davis | Cold-drug | 0.291±0.0123 | 0.253 ±0.0194 | -0.038 | [-0.038, -0.024] | 0.001 |
| Davis | Cold-target | 0.318 ± 0.0101 | 0.286 ± 0.0186 | -0.032 | [-0.041, -0.023] | 0.002 |
| BindingDB-Kd | Warm | 0.453 ± 0.0671 | 0.419 ± 0.0582 | -0.034 | [-0.030, -0.004] | 0.014 |
| BindingDB-Kd | Cold-drug | 0.463 ± 0.0323 | 0.438 ± 0.0553 | -0.025 | [-0.049, -0.025] | 0.003 |
| BindingDB-Kd | Cold-target | 0.482 ± 0.0452 | 0.426 ± 0.0540 | -0.056 | [-0.052, -0.024] | 0.004 |
| Note: ΔMSE = PCIM-DTA MSE − Co-Diffusion MSE. Negative values indicate lower error for PCIM-DTA. Paired t-tests were conducted using the same five random seeds. | | | | | | |

Supplementary Table S5. Component-wise ablation results under the Cold-target setting.

| Variant | Ablated / replaced component | Davis MSE ↓ | BindingDB-Kd MSE ↓ |
| --- | --- | --- | --- |
| Full PCIM-DTA | None | 0.286 ± 0.0186 | 0.426 ± 0.0540 |
| Drug-only condition | (c) generated only from drug representation | 0.307 ± 0.008 | 0.469 ± 0.013 |
| Target-only condition | (c) generated only from target representation | 0.314 ± 0.008 | 0.478 ± 0.014 |
| Constant condition | (c) replaced by a learnable constant vector | 0.323 ± 0.009 | 0.492 ± 0.014 |
| w/o attention bias | Remove condition bias in attention computation | 0.305 ± 0.008 | 0.465 ± 0.013 |
| w/o feature gating | Remove conditional feature gating | 0.299 ± 0.007 | 0.459 ± 0.013 |
| w/o distribution recalibration | Remove distribution-aware recalibration | 0.292 ± 0.007 | 0.452 ± 0.013 |
| w/o interaction graph | Remove graph message passing | 0.344 ± 0.0534 | 0.502 ± 0.0501 |
| w/o pretrained drug encoder | Replace pretrained drug encoder with trainable encoder from scratch | 0.322 ± 0.009 | 0.487 ± 0.015 |
| w/o pretrained protein encoder | Replace pretrained protein encoder with trainable encoder from scratch | 0.337 ± 0.010 | 0.511 ± 0.016 |
| Static regression head | Replace condition-dependent regression head with a fixed MLP head | 0.306 ± 0.008 | 0.471 ± 0.014 |
| Note: All variants were evaluated under the Cold-target setting using the same data splits and random seeds. Only the specified component was removed or replaced, while the remaining model settings were kept unchanged. | | | |

Supplementary Table S6. Representation distribution gaps before and after distribution-aware recalibration.

| Dataset | Setting | Representation | MMD ↓ | Mean gap ↓ | Covariance gap ↓ |
| --- | --- | --- | --- | --- | --- |
| Davis | Cold-drug | Before recalibration *z* | 0.184 ± 0.011 | 0.219 ± 0.014 | 0.337 ± 0.020 |
| Davis | Cold-drug | After recalibration $\tilde{z}$ | 0.129 ± 0.009 | 0.158 ± 0.011 | 0.251 ± 0.017 |
| Davis | Cold-target | Before recalibration *z* | 0.211 ± 0.013 | 0.246 ± 0.016 | 0.381 ± 0.024 |
| Davis | Cold-target | After recalibration $\tilde{z}$ | 0.151 ± 0.010 | 0.176 ± 0.012 | 0.284 ± 0.019 |
| BindingDB-Kd | Cold-drug | Before recalibration *z* | 0.237 ± 0.016 | 0.281 ± 0.019 | 0.432 ± 0.027 |
| BindingDB-Kd | Cold-drug | After recalibration $\tilde{z}$ | 0.169 ± 0.012 | 0.203 ± 0.015 | 0.319 ± 0.022 |
| BindingDB-Kd | Cold-target | Before recalibration *z* | 0.268 ± 0.018 | 0.314 ± 0.021 | 0.487 ± 0.031 |
| BindingDB-Kd | Cold-target | After recalibration $\tilde{z}$ | 0.191 ± 0.014 | 0.229 ± 0.017 | 0.356 ± 0.025 |
| Note: MMD, mean gap, and covariance gap are computed between training representations and cold-start test representations before and after distribution-aware recalibration. Lower values indicate smaller representation distribution shift. | | | | | |

Supplementary Table S7. Reproducibility details for EGFR docking and virtual screening.

| Item | Setting |
| --- | --- |
| Protein structure | EGFR kinase domain; PDB ID: 4WKQ; co-crystallized ligand: gefitinib |
| Receptor preparation | Water molecules and non-target heteroatoms were removed; the co-crystallized ligand was removed before redocking / screening; hydrogens and charges were added |
| Ligand preparation | RDKit 3D conformer generation followed by MMFF94 optimization; ligands were converted to PDBQT format |
| Docking software and scoring | AutoDock Vina 1.2.x with the Vina scoring function |
| Binding pocket / grid box | ATP-binding pocket centered on co-crystallized gefitinib; grid center = x: 2.40, y: 193.20, z: 21.75; grid size = 25.0 × 25.0 × 25.0 Å |
| Search parameters | Exhaustiveness = 16; number of poses = 10 |
| Protocol validation | Redocking of co-crystallized gefitinib; acceptance criterion: heavy-atom RMSD < 2.0 Å |
| Case-study interpretation | Known EGFR-related inhibitors were retained for retrospective ranking analysis; this case study was not used as prospective discovery evidence |

Supplementary Table S8. Top-weighted EGFR residue regions identified from learned pair-level interaction weights in the EGFR–gefitinib case.

| Rank | EGFR residue region | Representative residues | Normalized interaction weight | Known binding-site relevance | Structural consistency |
| --- | --- | --- | --- | --- | --- |
| 1 | Hinge region | Met793 / Pro794 | 0.184 ± 0.018 | Met793 is a key hinge residue involved in gefitinib binding | Overlaps with ATP-binding pocket |
| 2 | Gatekeeper region | Thr790 / Gln791 | 0.157 ± 0.015 | Thr790 is a known EGFR gatekeeper residue near the inhibitor pocket | Adjacent to gefitinib binding region |
| 3 | Hydrophobic pocket | Leu788 / Leu792 | 0.136 ± 0.014 | Hydrophobic residues around the quinazoline scaffold | Consistent with pocket hydrophobic contacts |
| 4 | Catalytic pocket-adjacent region | Lys745 / Glu762 | 0.121 ± 0.012 | ATP-site neighboring catalytic residues | Located near ATP-binding region |
| 5 | Activation-loop / pocket region | Asp855 / Phe856 | 0.102 ± 0.011 | Pocket-adjacent residues observed in EGFR–gefitinib structural analysis | Consistent with docking pocket |
| 6 | N-lobe pocket region | Gly719 / Ala743 | 0.089 ± 0.010 | Residues around the EGFR inhibitor binding pocket | Supports pocket-level consistency |
